# Supplementary material for: Longitudinal functional imaging of VIP interneurons reveals sup-population specific effects of stroke that are rescued with chemogenetic therapy
Source: Nat Commun. 2021 Oct 20;12:6112. doi: 10.1038/s41467-021-26405-6 (PMC8528851; doi:10.1038/s41467-021-26405-6)
Supplement: Supplementary file 1 — Supplementary information [file 41467_2021_26405_MOESM1_ESM.pdf]

Supplementary Information to "Longitudinal functional imaging of VIP interneurons reveals sup-population specific effects of stroke that are rescued with chemogenetic therapy"

Supplementary Figure 1

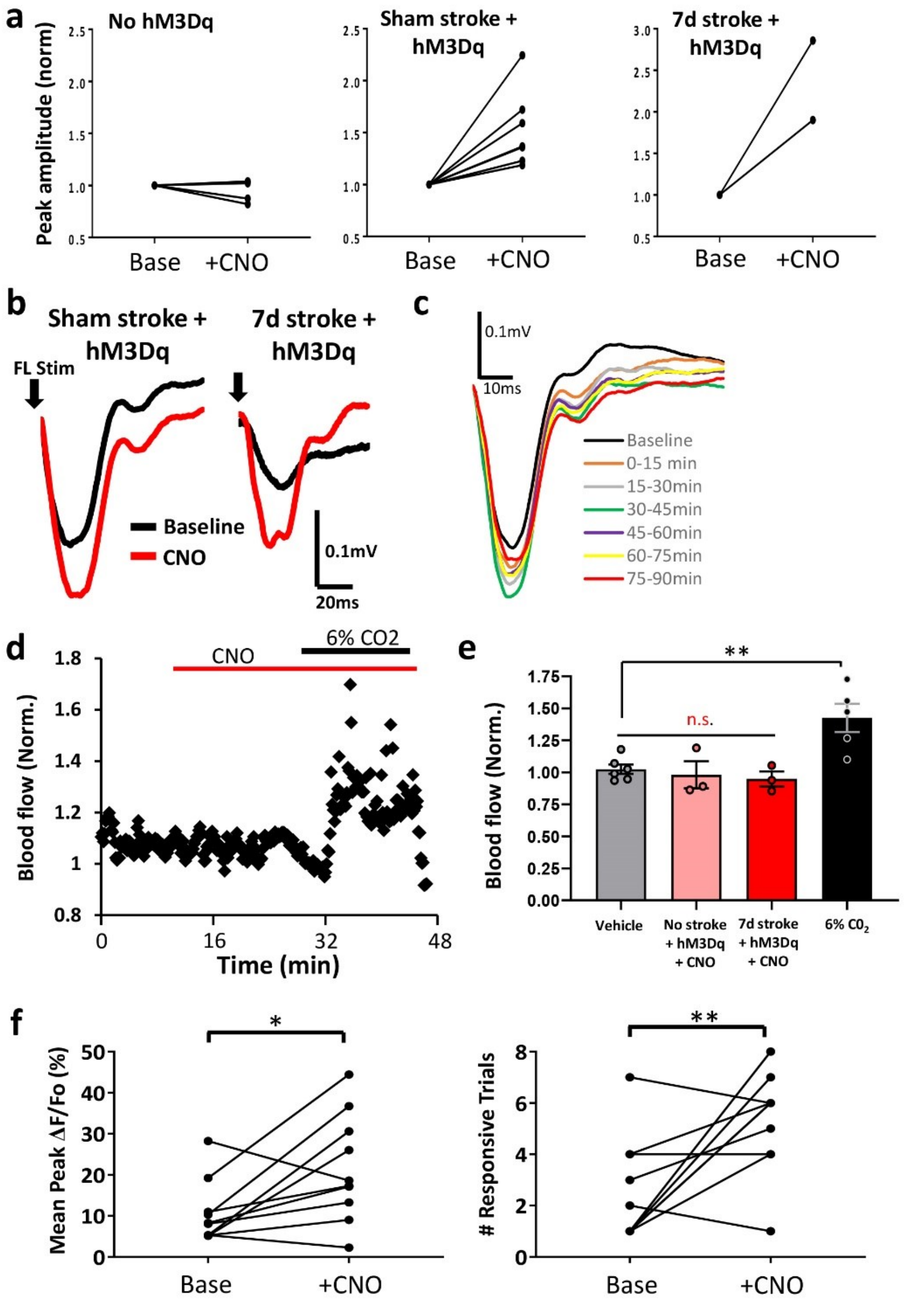

Supplementary Figure 1. CNO-hM3Dq activation enhances forelimb evoked cortical and VIP neuron responses but does not alter regional cerebral blood flow. (**a**) Change in peak forelimb evoked field potential after CNO treatment for individual mice in each experimental condition (n=5

No hM3Dq; n=7 sham stroke + hM3Dq; n=2 stroke + hM3Dq). (**b**) Group averaged forelimb-evoked responses at baseline and following CNO injection for both sham and stroke mice. (**c**) A representative field recording showing the effects of CNO injection (0.5mg/kg, i.p.) on forelimb evoked cortical responses over time. (**d**) Representative laser doppler flowmetry experiment showing regional blood flow in hM3Dq expressing mouse injected with CNO first and then exposed to 6% CO_2 which served as a positive control. (**e**) Bar graph shows that blood flow did not change significantly after vehicle or CNO injection (n=6 mice/group; 1-way ANOVA, $F_{(2,9)}=0.43$, p=0.66) but did when mice were exposed to 6% CO_2 (n=5 mice; unpaired t-test, $t_{(8)}=3.32$, p=0.005). (**f**) Injection of 0.5mg/kg CNO increased mean peak amplitude (left; Wilcoxon one-sided paired t-test, p=0.019) and # forelimb responsive trials (right; Wilcoxon one-sided paired t-test, p=0.008). Data collected from 10 GCaMP6s expressing VIP neurons from stroke recovered mice (n=3 mice at 14 weeks) with confirmed hM3Dq expression. **p<0.01, *p<0.05, n.s. = not significant. Data show means ± S.E.M.

Supplementary Figure 2

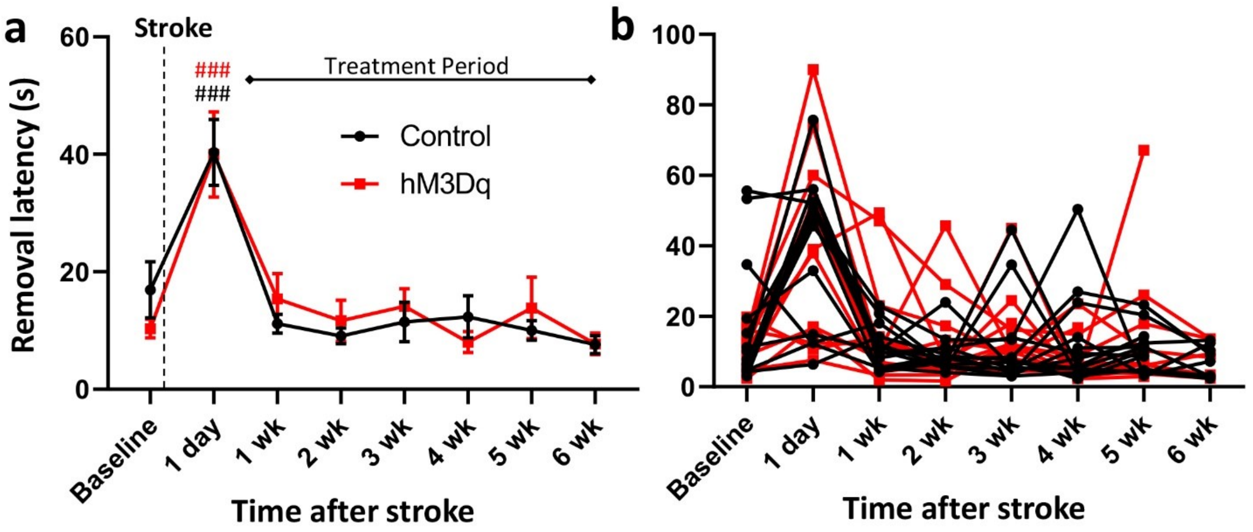

Supplementary Figure 2. Adhesive tape test was not sensitive for detecting longer term sensorimotor deficits after stroke. (**a**) Graph shows the average time it took mice to remove adhesive tape from the stroke affected (left) forepaw in mice receiving control (n=14 mice) or chemogenetic treatment (n=15 mice). Although stroke could induce a deficit at 1 day, no impairments were observed afterwards. (**b**) Plots show individual data points for mice that received control treatment (black lines) versus those that received chronic chemogenetic stimulation (red lines). ###$p<0.0001$ for two-sided t-test comparisons against baseline (pre-stroke). Data show means ± S.E.M.

Supplementary Figure 3

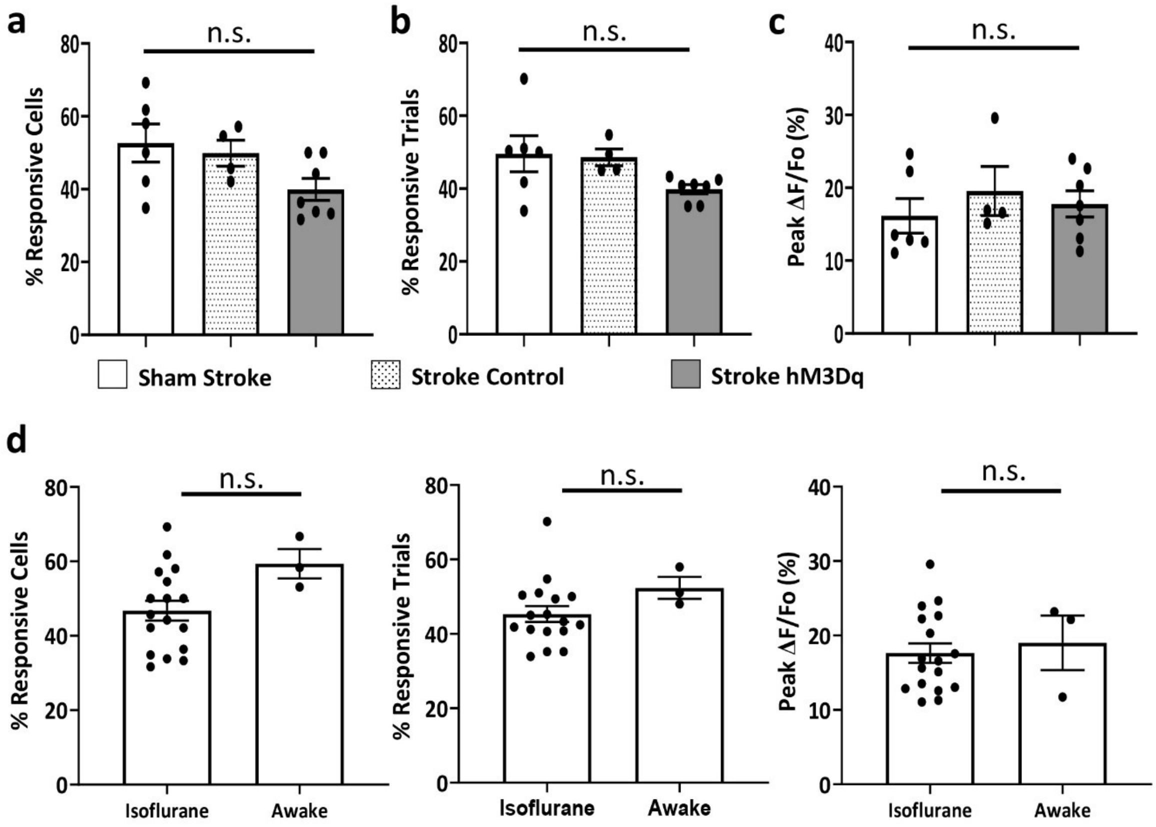

Supplementary Figure 3. Comparison of forelimb evoked responses in VIP neurons in anesthetized and awake mice. (a-c) Graphs show data for each mouse and average % forelimb responsive cells, % responsive trials and peak response amplitudes in peri-infarct cortex (<400µm of border) at baseline in the 3 experimental groups. (**d**) Graphs show data for each mouse and average % responsive cells (left), % responsive trials (middle) and mean peak response amplitude (right) in isoflurane anesthetized (17 mice from 3 experimental groups) and awake (3 mice) mice without stroke. One-way ANOVA (a-c) or unpaired two-sided student t-tests (d) did not reveal significant group differences. n.s. = not significant. Data show individual and group means ± S.E.M.

Supplementary Figure 4

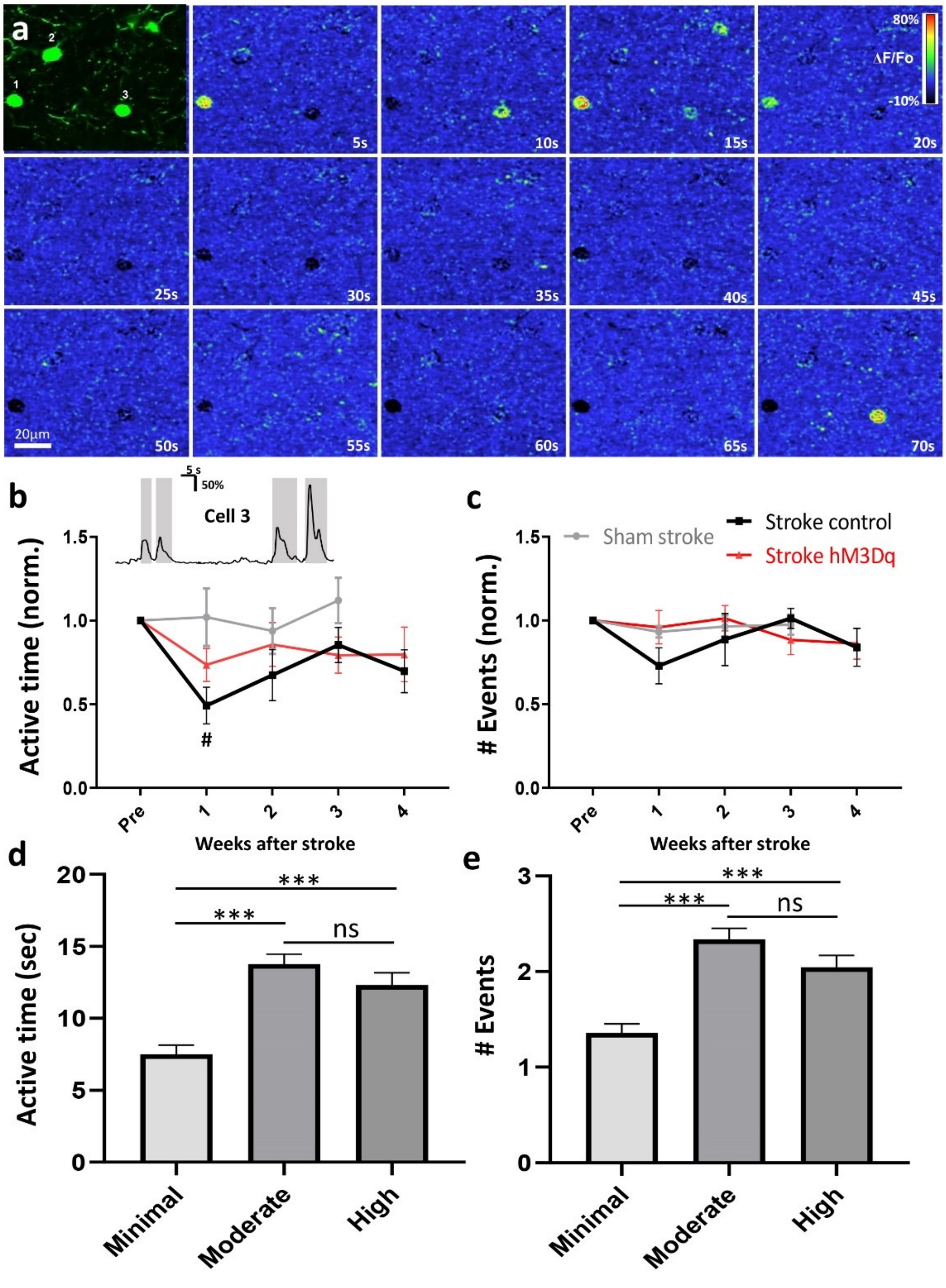

Supplementary Figure 4. Spontaneous cortical activity across experimental groups and within different VIP subpopulations. (**a**) Montages show spontaneous changes in GCaMP6s fluorescence (ΔF/Fo) in VIP neurons imaged over 70s period. (**b**) Top: traces extracted from cell 3 show spontaneous calcium events and the duration of each event (shaded grey). Bottom: graph plots relative amount of time neurons were active after stroke, normalized to pre-stroke. There was a significant

reduction in active time at week 1 relative to baseline for stroke mice that received control stimulation (n=6 mice; unpaired two-sided t-test, p=0.011), whereas no change was detected in sham stroke (n=4 mice) or stroke mice that received chemogenetic stimulation (n=7 mice). (**c**) Graph shows the number of spontaneous events occurring in VIP neurons after stroke normalized to pre-stroke. (**d,e**) There were significant differences in the average activate time per neuron (over 75 s period; 1-way ANOVA, $F_{(2,456)}$=23.28, p<0.0001) and number of spontaneous events (over 75s period; 1-way ANOVA, $F_{(2,456)}$=22.78, p<0.0001) across neurons classified as high (n=118 neurons), moderate (n=167 neurons) or minimally (n=175 neurons) responsive to forelimb stimulation. #p<0.05 for comparisons against baseline (pre-stroke). ***p<0.001 based on two-sided unpaired t-test. n.s. = not significant. Data show means ± S.E.M.

Supplementary Figure 5

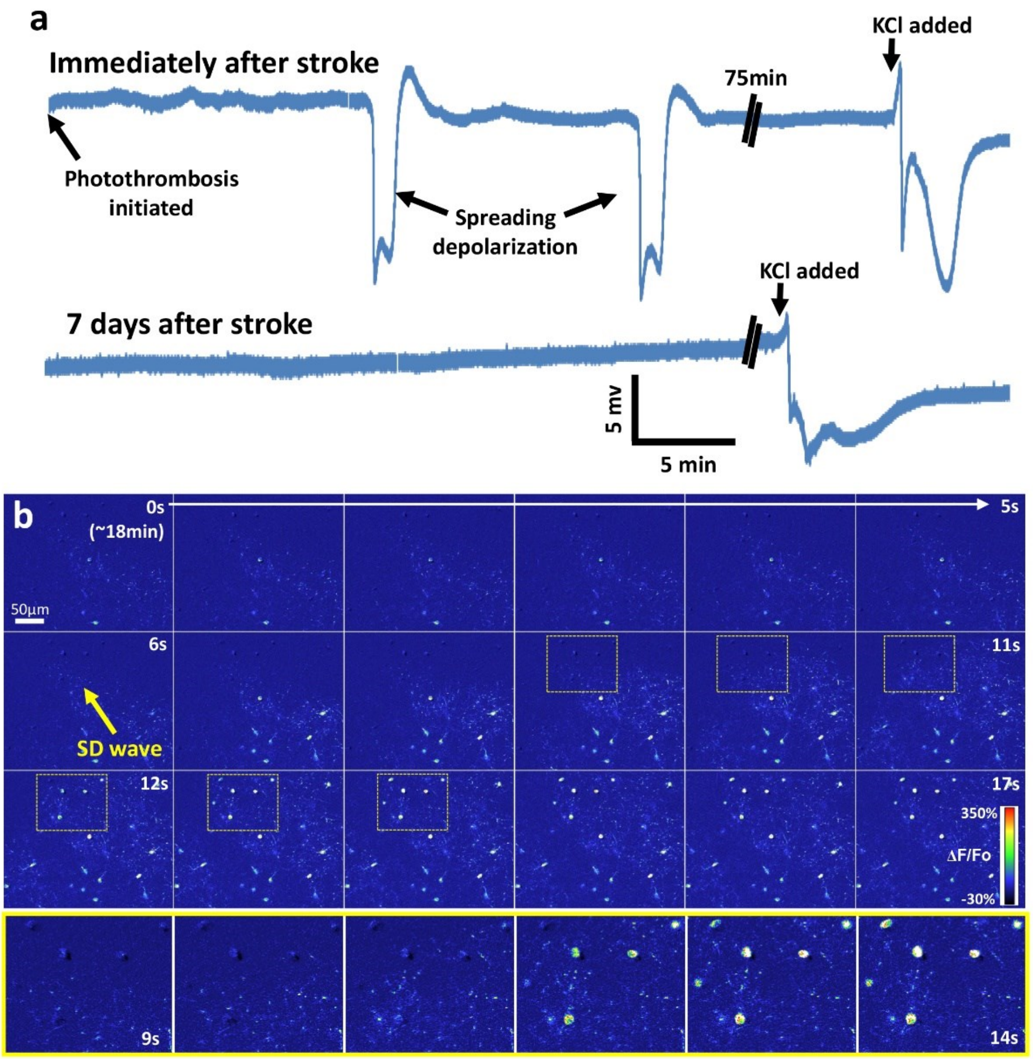

Supplementary Figure 5. Spreading depolarizations occurred in the first 30 min after photothrombotic stroke but not at 7 days recovery. (**a**) Representative examples of DC potentials recorded in peri-infarct cortex within minutes after the induction of photothrombotic stroke or at 7 days recovery (n=3 mice per group). We observed 1 or 2 spreading depolarizations within the first 30min after stroke in each animal but never at 7 days. Topical application of 1mM KCl was used as a positive control for inducing and detecting spreading depolarization waves. (**b**) Representative time-lapse 2-photon images collected at 1Hz starting ~18min after the induction of stroke show a large amplitude spreading depolarization wave propagating outward from cortex most proximal to stroke border (located in bottom right corner). Image below shows spreading depolarization wave propagated across the zoomed in region denoted with a dashed yellow box. These waves were never observed in peri-infarct cortex 7 days after stroke (n=3 mice).

Supplementary Figure 6

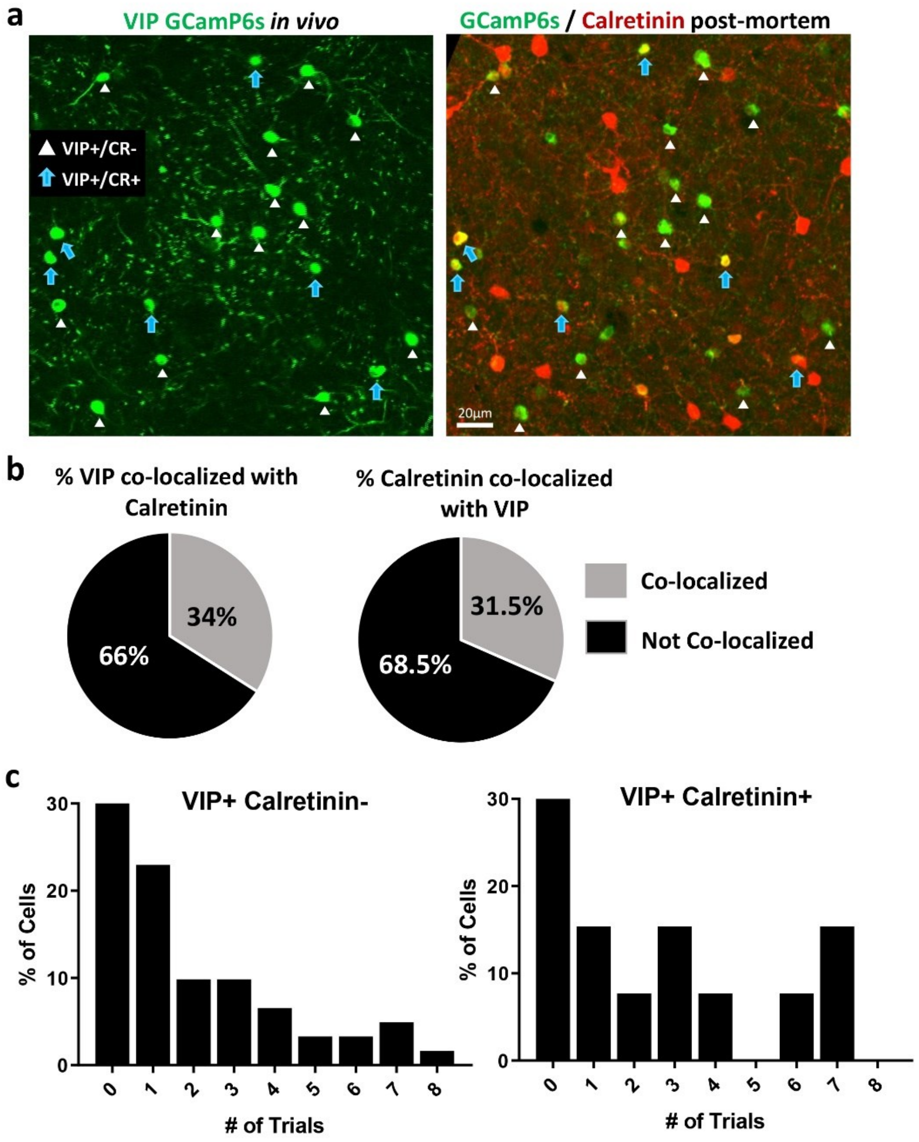

Supplementary Figure 6. Characterizing the response profile of VIP neurons that co-express calretinin (CR). (**a**) Representative example showing GCaMP6s expressing VIP neurons imaged *in vivo* (left panel) that were re-located in post mortem brain sections immunostained for CR (right panel). VIP neurons imaged *in vivo* that also expressed CR are indicated with cyan arrow, whereas those VIP neurons that did not express CR are denoted with white arrowhead. (**b**) Pie charts show % VIP neurons that co-localize with CR (385 VIP neurons examined) and % CR neurons that co-localize with VIP (415 CR neurons examined). (**c**) Histogram characterizing the forelimb response fidelity of VIP neurons that co-localize with CR or not. Data based on 2 mice: 71 neurons were VIP+/CR- and 14 neurons were VIP+/CR+.
