# Supplementary material for: Longitudinal functional imaging of VIP interneurons reveals sup-population specific effects of stroke that are rescued with chemogenetic therapy
Source: Nat Commun. 2021 Oct 20;12:6112. doi: 10.1038/s41467-021-26405-6 (PMC8528851; doi:10.1038/s41467-021-26405-6)
Supplement: Supplementary file 2 — Reporting Summary [file 41467_2021_26405_MOESM2_ESM.pdf]

nature portfolio

Corresponding author(s): Craig E. Brown

Last updated by author(s): Sep 21, 2021

Reporting Summary

Nature Portfolio wishes to improve the reproducibility of the work that we publish. This form provides structure for consistency and transparency in reporting. For further information on Nature Portfolio policies, see our Editorial Policies and the Editorial Policy Checklist.

Statistics

For all statistical analyses, confirm that the following items are present in the figure legend, table legend, main text, or Methods section.

n/a	Confirmed	
☐	☒	The exact sample size (n) for each experimental group/condition, given as a discrete number and unit of measurement
☐	☒	A statement on whether measurements were taken from distinct samples or whether the same sample was measured repeatedly
☐	☒	The statistical test(s) used AND whether they are one- or two-sided<br>*Only common tests should be described solely by name; describe more complex techniques in the Methods section.*
☐	☒	A description of all covariates tested
☐	☒	A description of any assumptions or corrections, such as tests of normality and adjustment for multiple comparisons
☐	☒	A full description of the statistical parameters including central tendency (e.g. means) or other basic estimates (e.g. regression coefficient) AND variation (e.g. standard deviation) or associated estimates of uncertainty (e.g. confidence intervals)
☐	☒	For null hypothesis testing, the test statistic (e.g. F, t, r) with confidence intervals, effect sizes, degrees of freedom and P value noted<br>*Give P values as exact values whenever suitable.*
☒	☐	For Bayesian analysis, information on the choice of priors and Markov chain Monte Carlo settings
☒	☐	For hierarchical and complex designs, identification of the appropriate level for tests and full reporting of outcomes
☒	☐	Estimates of effect sizes (e.g. Cohen's d, Pearson's r), indicating how they were calculated

Our web collection on statistics for biologists contains articles on many of the points above.

Software and code

Policy information about availability of computer code

Data collection	Olympus FluoView (FV1000-Version03.01);<br>Brain Vision BV-Ana(PCI) X86 edition (Version 12.08.20);<br>Clampex (Version10.2.0.12);<br>Q-Capture Pro 7;<br>MoorVMS-pc version 2.2;
Data analysis	ImageJ/FIJI v1.52p (Java1.8.0_172 - 64bit) ;<br>ImageJ/FIJI plug-ins StackReg and TurboReg (Version: July 7, 2011)<br>http://bigwww.epfl.ch/thevenaz/turboreg/;<br>http://bigwww.epfl.ch/thevenaz/stackreg/;<br>Microsoft Excel 2016;<br>GraphPad Prism 8 software (version 8.4.3);

For manuscripts utilizing custom algorithms or software that are central to the research but not yet described in published literature, software must be made available to editors and reviewers. We strongly encourage code deposition in a community repository (e.g. GitHub). See the Nature Portfolio guidelines for submitting code & software for further information.

Data

Policy information about availability of data

All manuscripts must include a data availability statement. This statement should provide the following information, where applicable:

- Accession codes, unique identifiers, or web links for publicly available datasets
- A description of any restrictions on data availability
- For clinical datasets or third party data, please ensure that the statement adheres to our policy

Data generated for this study are available from the corresponding author on reasonable request. Source data are provided with this paper.

Field-specific reporting

Please select the one below that is the best fit for your research. If you are not sure, read the appropriate sections before making your selection.

- [x] Life sciences
- [ ] Behavioural & social sciences
- [ ] Ecological, evolutionary & environmental sciences

For a reference copy of the document with all sections, see nature.com/documents/nr-reporting-summary-flat.pdf

Life sciences study design

All studies must disclose on these points even when the disclosure is negative.

Sample size	Sample sizes were based on comparable n-values from previously published literature (see refs 7,32,33,43,44).
Data exclusions	Four weeks after surgical implantation of glass cranial windows, the quality of imaging windows was examined and mice that had windows with a significant loss of clarity were excluded from the study.<br>Given the longitudinal nature of calcium imaging study (imaging sensory evoked responses and spontaneous activity), cells that were not identifiable over weeks were excluded from the analysis.
Replication	Data was collected from two or three experimental groups including Stroke control; Stroke hM3Dq; Sham stroke. Each experimental group consisted of multiple animal subjects. Behavioural data were collected in 2 large cohorts by 2 different experimenters. The first cohort was run by K.G., which was later replicated by S.C. Similarly, the effects of DREADD stimulation on cortical field potentials and blood flow were first described by K.G, and then later replicated by M.M. For calcium imaging, all experiments were collected and analyzed by M.M. Experimental and control mice were run in parallel in small cohorts (1-2 mice/group), which were later repeated and replicated to arrive at 4-7 mice per group. All replication attempts were successful.
Randomization	Mouse litter mates were randomly assigned (flip of the coin) to experimental or control conditions and run in parallel to avoid any litter or batch effects.
Blinding	Behavioural data was collected by an experimenter blind to condition. Experimenters were not blinded during data collection for calcium imaging, Voltage sensitive (VSD) imaging, and recording of cortical field potentials, since the experimenter needed to determine the location of the infarct for proper data collection (imaging or recording). However, data analysis for behavioural data, histology, calcium imaging, voltage sensitive dye (VSD) imaging, and cortical field potentials was performed using pre-determined algorithms that minimize any subjective bias. Further, researchers were blind to condition. For blood flow imaging experiments, our experimental design was predetermined (i.p. injection of saline, then CNO, then CO2), therefore the experimenter could not be blind to the injection. However, our analysis of blood flow was based on pre-defined algorithms that would treat data sets from different groups the exact same and therefore minimize any potential for experimenter induced bias.

Reporting for specific materials, systems and methods

We require information from authors about some types of materials, experimental systems and methods used in many studies. Here, indicate whether each material, system or method listed is relevant to your study. If you are not sure if a list item applies to your research, read the appropriate section before selecting a response.

Materials & experimental systems

n/a	Involved in the study	
[ ]	[x]	Antibodies
[x]	[ ]	Eukaryotic cell lines
[x]	[ ]	Palaeontology and archaeology
[ ]	[x]	Animals and other organisms
[x]	[ ]	Human research participants
[x]	[ ]	Clinical data
[x]	[ ]	Dual use research of concern

Methods

n/a	Involved in the study	
[x]	[ ]	ChIP-seq
[x]	[ ]	Flow cytometry
[x]	[ ]	MRI-based neuroimaging

Antibodies

Antibodies used	Novus pAb (polyclonal) Rabbit anti-mCherry (Novus Biologicals NBP2-25157, lot # 010519) used at 1:1000; Rabbit anti-calretinin (Sigma, AB5054) used at 1:2000.<br>Invitrogen by Thermo Fisher Scientific Cy5 (polyclonal) Goat anti Rabbit IgG (H+L) cross-adsorbed secondary antibody, Cyanine5 (Invitrogen A10523 , lot # 1917948) used at 1:500 ;<br>Cy5 conjugated Goat anti-Rabbit cross-adsorbed secondary antibody (Invitrogen A10523; dilution 1:500)
Validation	lease refer to the manufacturer's website for a list of publications: https://www.novusbio.com/products/mcherry-antibody_nbp2-25157<br>This mCherry antibody is useful for Immunocytochemistry/Immunofluorescence and Western Blot, where a band can be seen at ~28 kDa.<br>Use in IHC and IHC-P reported in scientific literature (PMID: 27396338 and 28891816 respectively).<br>Use in Live Imaging Microscopy was reported from a verified customer review.<br>Knockdown validation (PMID: 32494070).<br>Use in Chromatin Immunoprecipitation was reported in scientific literature (PMID: 28934387).<br>The Calretinin antibody (Sigma, AB5054) has been validated for use in immunohistochemistry and western blot. https://www.sigmaaldrich.com/CA/en/product/mm/ab5054?context=product

Animals and other organisms

Policy information about studies involving animals; ARRIVE guidelines recommended for reporting animal research

Laboratory animals	Two to five-month-old male and female VIP-IRES-cre mice (Jackson Laboratory, stock no. 010908) were used in this study. Room temperature was kept between 21-24ºC and between 40-60% relative humidity
Wild animals	The study did not involve wild animals.
Field-collected samples	The study did not involve samples collected from the field.
Ethics oversight	All experiments were conducted in accordance with the guidelines laid out by the Canadian Council of Animal Care and approved by the University of Victoria Animal Care Committee.

Note that full information on the approval of the study protocol must also be provided in the manuscript.
